# Persistence of environmental DNA in marine systems

**DOI:** 10.1038/s42003-018-0192-6

**Published:** 2018-11-05

**Authors:** Rupert A. Collins, Owen S. Wangensteen, Eoin J. O’Gorman, Stefano Mariani, David W. Sims, Martin J. Genner

**Affiliations:** 1 School of Biological Sciences, University of Bristol, Life Sciences Building, Tyndall Avenue, Bristol, BS8 1TQ UK; 2 Ecosystems & Environment Research Centre, School of Environment & Life Sciences, University of Salford, Salford, M5 4WT UK; 3 Norwegian College of Fishery Science, UiT The Arctic University of Norway, Tromsø, N-9037 Norway; 4 Imperial College London, Silwood Park Campus, Buckhurst Road, Ascot, Berkshire, SL5 7PY UK; 5 Marine Biological Association of the United Kingdom, The Laboratory, Citadel Hill, Plymouth, PL1 2PB UK; 6 Ocean and Earth Science, University of Southampton, National Oceanography Centre Southampton, European Way, Southampton, SO14 3ZH UK

## Abstract

As environmental DNA (eDNA) becomes an increasingly valuable resource for marine ecosystem monitoring, understanding variation in its persistence across contrasting environments is critical. Here, we quantify the breakdown of macrobial eDNA over a spatio-temporal axis of locally extreme conditions, varying from ocean-influenced offshore to urban-inshore, and between winter and summer. We report that eDNA degrades 1.6 times faster in the inshore environment than the offshore environment, but contrary to expectation we find no difference over season. Analysis of environmental covariables show a spatial gradient of salinity and a temporal gradient of pH, with salinity—or the biotic correlates thereof—most important. Based on our estimated inshore eDNA half-life and naturally occurring eDNA concentrations, we estimate that eDNA may be detected for around 48 h, offering potential to collect ecological community data of high local fidelity. We conclude by placing these results in the context of previously published eDNA decay rates.

## Abstract (web summary)

Rupert A. Collins et al. show that environmental DNA degrades faster in the inshore urban environment than the ocean-influenced offshore environment. This study suggests that environmental DNA can be reliably detected for two days, providing an optimal time window of high local fidelity.

## Introduction

The ability to sequence minute concentrations of extra-organismal DNA directly from the aquatic environment is transforming ecological monitoring and environmental management^1–3^. However, the reliability and resolution of our inferences from these environmental DNA (eDNA) surveys is contingent upon the ability to detect the contemporaneous presence of a species, or provide an accurate representation of a community at a specific point in time. The duration or persistence of eDNA molecules in the environment is therefore of critical importance^4,5^. For example, comparisons of species richness across protected areas^6^ or along ecological gradients^7^ require consideration of two possibilities. Firstly, that species that are present may not be detected due to, for example, low-organism density (a false negative), or secondly, that species currently absent or never present are detected due to eDNA being transported in from connected areas (false positive). Knowledge of how long eDNA is likely to persist in a given system is therefore of importance to understanding both of these scenarios, and is a pertinent problem for eDNA studies of lotic and marine ecosystems in particular, due to the potential influence of eDNA transport via river or tidal currents. Deiner et al.^8^, for instance, reported that eDNA could be recovered up to 10 km downstream of a source population, while Kelly et al.^9^ reconstructed site-specific communities despite a tidal cycle.

To date, the majority of studies on eDNA degradation rates have focused on freshwater habitats, and mainly in terms of simulated lentic environments in mesocosm experiments, and often using non-natural water sources^10–13^. Experiments representing more diverse natural systems and conditions are now being conducted, for example in ponds with different nutrient profiles^14^, or in stream mesocosms across a natural acid–base gradient^5^. In the marine environment, most studies of eDNA degradation have been preliminary or as supporting evidence in wider metabarcoding studies^15–19^. Sassoubre et al.^20^, however, made a detailed comparison of release and decay rates among marine fish species, while Andruszkiewicz et al.^21^ and Jo et al.^22^ investigated the effects of ultraviolet light and fragment length on marine eDNA decay rates, respectively. Microbiologists have undertaken degradation studies with DNA from marine bacteria typical of faecal pollution events^23–25^, but it is unclear if these can be generalised due to the differences between prokaryotic and eukaryotic cells.

Marine systems present a different set of conditions to freshwater systems in terms of eDNA stability, and previous studies have suggested that eDNA degrades faster in marine systems^18,20^, despite the potential preservative effect of salt on DNA^23^. Differences in chemical composition, pH, temperature and biota all play an important role in freshwater eDNA dynamics, with warmer water of a neutral or acidic pH and a low dissolved organic carbon content having the highest degradation rates^5,12,14^. However, despite being more chemically homogeneous than freshwater, heterogeneity in natural seawater taken from different locations or at different times of the year has yet to be fully explored (but for a microbial perspective on seasonal nutrient limitation and organic phosphorus, see Salter^26^).

Here, we evaluate the influence of season and location on eDNA degradation rates by collecting water from different environments in the Western English Channel, representing putatively extreme regional conditions that differ chemically and biologically, and where differential decay may be expected^14,26^, viz., an unstable inshore–urban location with high levels of anthropogenic and freshwater terrestrial inputs, a stable, seasonally stratified offshore site beyond the frontal isotherm representing ocean-influenced conditions, and a simulated environmental gradient created by mixing water from these two locations. Experimental water was spiked with natural eDNA from two common European intertidal species (fish and crab). Temporal degradation in eDNA was measured by quantitative PCR (qPCR) in a controlled aquarium laboratory setup. The experiment was repeated over two contrasting seasons, late winter and late summer, when sea surface temperatures and primary production should be near their respective minima and maxima in this region^27^. We hypothesise, firstly, that the inshore site will show a faster degradation rate than the offshore site due to a wider range of potential factors that may influence degradation (e.g. freshwater input, lower pH), and secondly, that the summer season will show a faster rate than winter due to the higher temperatures and increased biological activity. Our findings show, as predicted, that eDNA degrades faster in the inshore site than the offshore site, but contrary to our expectations, it is not possible to statistically distinguish summer decay rates from winter decay rates.

## Results

### Assay design and controls

A total of 18,675 *COI* (5′ mitochondrial cytochrome c oxidase I gene) sequences from 759 fish and malacostracan species were obtained from GenBank. Twelve *COI* sequences were obtained from our reference specimens. In silico PCR using MFEprimer indicated no off-target amplifications for the shanny (*Lipophrys pholis*) and common shore crab (*Carcinus maenas*) primer pairs chosen (Supplementary Table 1).

Mean assay efficiencies as reported from the standard curves on each plate were 103% (SD = 4.7) for the shanny assay and 106% (SD = 4.3) for the crab assay. Mean *R*^2^ values for both assays were 0.996 (SD = 0.004). At 1 μL of standard per reaction, the crab assay amplified 97% of the 10 copies/μL standards, and 37% of the 1 copy/μL standards. The shanny assay amplified 97% of the 10 copies/μL standards, and 30% of the 1 copy/μL standards. Following Agersnap et al.^28^, the limit of quantification for both assays was ~10 copies/μL (=833 copies/L) and the limit of detection was around 1 copy/μL (=83.3 copies/L). The highest Ct value for a reliable amplification was 38.5, and all positive amplifications below this value were used in subsequent analyses even if below the limit of quantification. In the winter experiment, the proportion of non-amplifying qPCR reactions was 0 at 96 h and 0.56 at 192 h; in the summer experiment, the proportion was 0.19 at 96 h and 0.68 at 192 h.

None of the no-template controls amplified in the multiplex qPCR assays. A total of 22 (12 shanny, 10 crab) of the 96 no-treatment controls amplified in one or more qPCRs, with 13 (4 shanny, 9 crab) of these (60%) from the inshore water control where these species were expected to occur. Of the amplifications not from the inshore control, all but two were in just one of the technical replicates, and the mean contamination level averaging over only the positive qPCRs was 70 copies/L (crab assay) and 186 copies/L (shanny assay).

Of the 24 DNA extractions tested for PCR inhibitors with serial dilution and qPCR, the mean efficiency value was 97% and the maximum was 111.3% (winter, offshore, tank 15).

### Persistence times

**Fig. 1:**
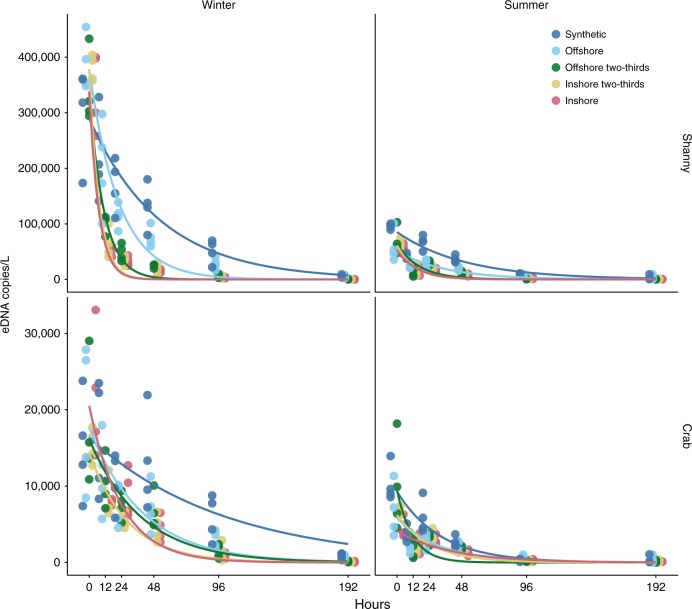
Exponential eDNA decay. Environmental DNA decay over 192 h, two seasons (summer and winter), two species (shanny and common shore crab assays) and five experimental water treatments simulating an environmental gradient. Response variable is eDNA concentration in copies per litre of treatment water. Zero hour data at *t* = 0 are included. Trend lines show an exponential decay model

**Fig. 2:**
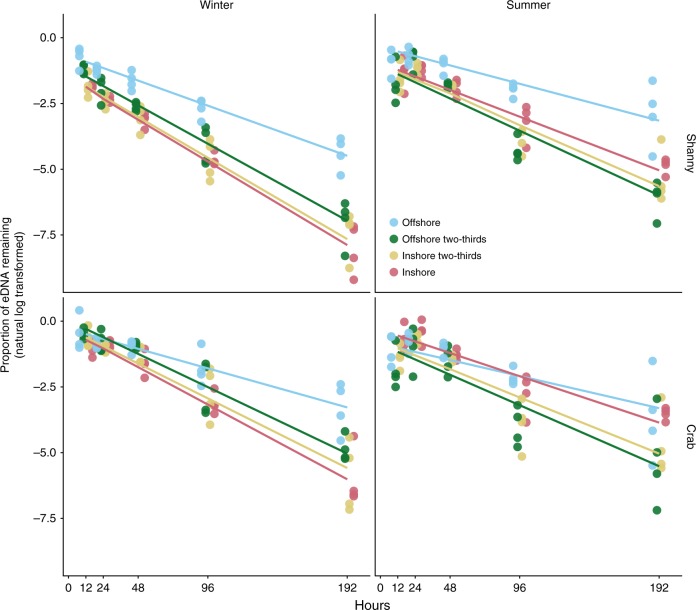
Rates of eDNA decay. Environmental DNA decay over 192 h, two seasons (summer and winter), two species (shanny and common shore crab assays) and four experimental water treatments simulating an environmental gradient. The response variable is natural log_*e*_ transformed eDNA concentration normalised as a proportion of starting concentration, i.e. the value at time *t* = *x* divided by the value at time *t* = 0. Zero hour data at *t* = 0 were subsequently excluded after proportions were calculated. Trend lines show fitted linear regression values from the optimal linear mixed-effects model

Over 192 h, eDNA showed an exponential decay in copies per litre of seawater over two seasons, two species and five experimental water treatments (Fig. 1; Fig. 2). The overall eDNA decay rate *k* across the natural treatments (synthetic control excluded) and seasons was −0.27, which translates to an eDNA half-life of 26.2 h (Table 1). The fastest decay rates were the inshore mixed treatments during the winter crab treatment (−0.033; 21.2 h), while the slowest rate was the offshore shanny treatment during the summer experiment at (−0.015; 45.6 h).

**Table 1:** Rates of eDNA decay

Water treatment	Season	Assay	Decay rate constant *k* [95% CI]	Hours $t_{\frac{1}{2}}$ [95% CI]
All	All	All	−0.027 [−0.023, −0.03]	26.2 [23.4, 29.7]
		Crab	−0.029 [−0.022, −0.035]	24.3 [19.8, 31.2]
		Shanny	−0.024 [−0.022, −0.027]	28.4 [26.1, 31]
	Summer	All	−0.025 [−0.02, −0.03]	27.5 [22.9, 34.5]
		Crab	−0.027 [−0.019, −0.036]	25.4 [19.5, 36.5]
		Shanny	−0.023 [−0.02, −0.026]	30 [26.6, 34.3]
	Winter	All	−0.028 [−0.025, −0.03]	24.9 [22.8, 27.4]
		Crab	−0.03 [−0.025, −0.035]	23.2 [19.9, 27.8]
		Shanny	−0.026 [−0.022, −0.029]	26.9 [23.6, 31.3]
Offshore	All	All	−0.019 [−0.014, −0.023]	37.3 [30.3, 48.5]
	Summer	Crab	−0.019 [−0.011, −0.028]	35.8 [24.7, 65.3]
		Shanny	−0.015 [−0.011, −0.019]	45.6 [35.9, 62.3]
	Winter	Crab	−0.022 [−0.016, −0.028]	31.6 [25, 42.8]
		Shanny	−0.018 [−0.013, −0.022]	38.9 [30.9, 52.5]
Offshore two-thirds	All	All	−0.029 [−0.025, −0.034]	23.6 [20.4, 28]
	Summer	Crab	−0.03 [−0.021, −0.039]	23 [17.8, 32.6]
		Shanny	−0.026 [−0.021, −0.03]	26.7 [22.7, 32.3]
	Winter	Crab	−0.033 [−0.027, −0.039]	21.2 [18, 25.8]
		Shanny	−0.029 [−0.024, −0.034]	24.3 [20.7, 29.3]
Inshore two-thirds	All	All	−0.029 [−0.025, −0.034]	23.6 [20.4, 27.9]
	Summer	Crab	−0.03 [−0.021, −0.039]	23 [17.8, 32.5]
		Shanny	−0.026 [−0.022, −0.03]	26.7 [22.8, 32]
	Winter	Crab	−0.033 [−0.027, −0.039]	21.2 [17.9, 25.8]
		Shanny	−0.029 [−0.024, −0.034]	24.2 [20.7, 29.3]
Inshore	All	All	−0.029 [−0.024, −0.033]	24.1 [21, 28.5]
	Summer	Crab	−0.029 [−0.021, −0.038]	23.5 [18.1, 33.6]
		Shanny	−0.025 [−0.021, −0.029]	27.4 [23.6, 32.6]
	Winter	Crab	−0.032 [−0.026, −0.038]	21.6 [18.3, 26.4]
		Shanny	−0.028 [−0.023, −0.032]	24.8 [21.4, 29.6]
Synthetic	All	All	−0.019 [−0.015, −0.022]	36.8 [31.2, 44.7]

Environmental DNA decay rate constant (*k*) and half-life $\left( {t_{\frac{1}{2}}} \right)$ over treatment, season and assay combinations, with 95% confidence intervals. Constants were estimated from the optimal linear mixed-effects model using the emtrends function in emmeans. Rates for the synthetic treatment were estimated from a separate model.

**Fig. 3:**
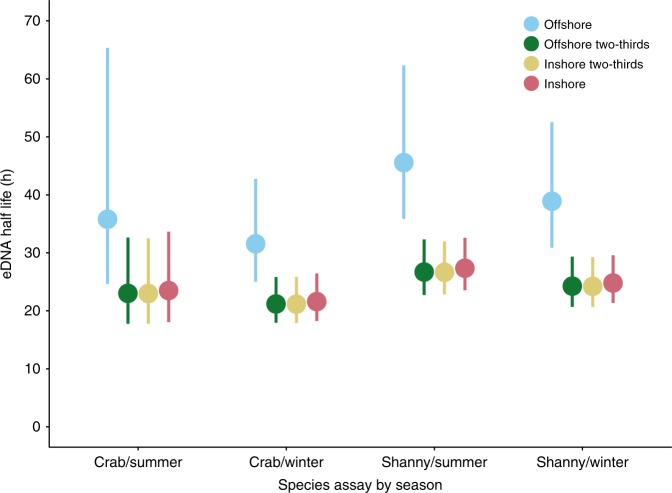
Half-life of eDNA. Environmental DNA half-lives (hours) for each water treatment and season–species combination. Half-lives were calculated from rate constants estimated from an optimal linear mixed-effects model using the emtrends function in emmeans. Dots represent point estimates derived from the model, with bars showing 95% confidence intervals also estimated by the model

Degradation rates were consistently slower—and therefore half-lives consistently longer—in the offshore water treatments than the inshore and the mixed offshore/inshore treatments, for both season and species (Fig. 2; Fig. 3; Table 1), and this was statistically significant (*p* < 0.0003; Table 2). There were no differences among the inshore and mixed treatments (*p* > 0.99; Table 2). The overall difference between the offshore and inshore treatments—i.e. averaged over assay and season—was 13.9 h (1.55 times slower offshore). Degradation rates were faster in the crab assay than the shanny assay by 4.1 h overall (1.17 times slower in the shanny), but this difference was not statistically significant (*p* = 0.25; Table 2). Overall degradation rates were faster in winter than in summer by 2.6 h (1.1 times slower in summer), and this was not statistically significant (*p* = 0.31; Table 2). Degradation rates in the synthetic control were most similar to the offshore treatment (−0.019; 36.8 h), and did not differ by assay or season (Table 1; Supplementary Fig. 1).

**Table 2:** Statistical comparisons

Predictor	Contrast 1	Contrast 2	Response estimate [SE]	*t* ratio	*p*-value
Season	Summer	Winter	0.003 [0.003]	1.022	0.3077
Assay	Crab	Shanny	−0.004 [0.004]	−1.162	0.2462
Treatment	Offshore	Inshore	0.01 [0.002]	4.171	0.0002
		Inshore two-thirds	0.011 [0.003]	4.212	0.0002
		Offshore two-thirds	0.011 [0.003]	4.134	0.0003
	Offshore two-thirds	Inshore	−0.001 [0.003]	−0.239	0.9952
		Inshore two-thirds	0 [0.003]	0.017	1
	Inshore	Inshore two-thirds	0.001 [0.003]	0.261	0.9938

*SE* standard error

Estimated marginal mean responses estimated from the optimal linear mixed-effects model using the emtrends function in emmeans. Responses are averaged over assay, season or treatment, according to contrast

### Environmental covariates

Environmental covariates are presented in Table 3. Overall, pH values were higher in summer than winter across the natural water treatments by an average of 0.49 units, while electrical conductivity (salinity) was lower by 0.7 mS/cm (1.3%). The offshore treatment had a higher pH than the inshore treatment by an average of 0.03 units, but conductivity was higher by 5.1 mS/cm (9%). Background DNA was lower in the offshore treatment (418 ng/L) than the inshore treatment (843 ng/L) in winter, but higher in the offshore (1475 ng/L) than the inshore treatment (240 ng/L) in summer. Temperature at collection in winter was 10.2 °C for offshore, 9.8 °C inshore, while in summer, it was 15.4 °C for offshore and 16.9 °C for inshore. The synthetic seawater control was characterised by low conductivity (winter 43.5 mS/cm, summer 43.1 mS/cm), high pH (winter 8.38, summer 8.77) and low background DNA (winter 45.6 ng/L, summer 102 ng/L).

**Table 3:** Environmental covariates

Season	Water treatment	bDNA	Mean pH [SD]	Mean EC [SD]
Summer	Synthetic	102.5	8.77 [0.03]	43.1 [0.05]
	Offshore	1475.0	8.56 [0.03]	55.4 [0.08]
	Offshore two-thirds		8.55 [0.02]	53.9 [0.24]
	Inshore two-thirds		8.5 [0.07]	52.6 [0.29]
	Inshore	239.7	8.53 [0.05]	51 [0.17]
Winter	Synthetic	45.6	8.38 [0.01]	43.5 [0.12]
	Offshore	417.5	8.06 [0.01]	56.5 [0.1]
	Offshore two-thirds		8.06 [0.03]	54.9 [0.13]
	Inshore two-thirds		8.05 [0.01]	53.2 [0.62]
	Inshore	843.3	8.04 [0.02]	50.8 [0.21]

*bDNA*  background DNA in copies/L (single value taken per season/treatment), *EC*  electrical conductivity/salinity (mS/cm), *SD* standard deviation

Environmental covariates from each tank replicate averaged over each season and water treatment combination.

Of the possible covariates, conductivity was found to negatively correlate with eDNA degradation (*p* = 0.0004), with pH and background DNA concentration having no detectable effect (*p* = 0.33; *p* = 0.93). Starting DNA concentration was significantly positively correlated with degradation (*p* < 0.0001). In a combined model, pH covaried better with season than treatment (0.96 vs. <0.3), while salinity covaried better with treatment than season (>0.93 vs. 0.08).

## Discussion

Our results show evidence for a strong spatial effect of eDNA degradation in the natural marine environment, with eDNA degrading around 1.6 times faster in the terrestrially influenced inshore environment than the ocean-influenced offshore environment. We found that eDNA also degraded around 1.1 times faster in winter than in summer, although this difference was not statistically significant.

These results placed in the context of our review of eDNA decay rates in the literature (Table 4), appear to contradict the notion that eDNA degrades faster in marine environments than freshwater^18,20,29^. In fact, degradation rates appear to be slower in many cases, with only marine studies or the freshwater studies at low temperature or using non-natural water sources, having a half-life of greater than 30 h (Table 4). The fastest rates in freshwater systems assessed so far are of acidic stream environments (<1.2 h of half-life^5^), while the fastest marine decay rate was 6.9 h, from anchovy eDNA in Californian inshore waters at 22 °C^20^. Most marine eDNA decay rates appear, however, to have been estimated at between 10 and 50 h, and with the lowest rates corresponding to the coldest water temperatures: 63 h at 4 °C^19^ and 71 h at −1 °C^15^. Rates above 71 h were from freshwater studies using sanitised or purified water from non-natural sources (Table 4).

**Table 4:** Literature review

Study	Organism	Environment	Water source	Fragment length (bp)	Temperature (°C)	pH	Half-life (h)
Seymour et al.^5^	Multi-species (fish/inverts)	Freshwater	Stream	100–132	16	5.3–5.8	0.7
Seymour et al.^5^	Multi-species (fish/inverts)	Freshwater	Stream	100–132	14	5.3–5.8	0.7
Seymour et al.^5^	Multi-species (fish/inverts)	Freshwater	Stream	100–132	15	6.8–7.2	1.0
Seymour et al.^5^	Multi-species (fish/inverts)	Freshwater	Stream	100–132	15	6.8–7.2	1.2
Tsuji et al.^31^	Ayu sweetfish	Freshwater	River	131	30	7.5	2.8
Tsuji et al.^31^	Common carp	Freshwater	River	78	30	7.5	2.8
Tsuji et al.^31^	Ayu sweetfish	Freshwater	River	131	20	7.5	4.9
Tsuji et al.^31^	Common carp	Freshwater	River	78	20	7.5	4.9
Barnes et al.^11^	Common carp	Freshwater	Well	146	25	7.5	6.6
Maruyama et al.^63^	Bluegill sunfish	Freshwater	Tap	100	20		6.7
Eichmiller et al.^14^	Common carp	Freshwater	Eutrophic lake	149	25		6.9
Sassoubre et al.^20^	Northern anchovy	Marine	Local inshore	133	22		6.9
Eichmiller et al.^14^	Common carp	Freshwater	Eutrophic lake	149	35		7.0
Eichmiller et al.^14^	Common carp	Freshwater	Oligotrophic lake	149	15		7.1
Jo et al.^22^	Japanese jack mackerel	Marine	Local inshore	719			7.7
Pilliod et al.^64^	Idaho giant salamander	Freshwater	Spring	84	11–25		8.8
Eichmiller et al.^14^	Common carp	Freshwater	Eutrophic lake	149	15		8.9
Eichmiller et al.^14^	Common carp	Freshwater	Eutrophic lake	149	15		9.8
Sassoubre et al.^20^	Pacific chub mackerel	Marine	Local inshore	107	19		9.9
Pilliod et al.^64^	Idaho giant salamander	Freshwater	Spring	84	13–20		10.1
Sassoubre et al.^20^	Pacific sardine	Marine	Local inshore	107	19		10.2
Sansom & Sassoubre^50^	Freshwater mussel	Freshwater	Tap	147	22		13.1
Jo et al.^22^	Japanese jack mackerel	Marine	Local inshore	127			15.8
Sigsgaard et al.^17^	Whale shark	Marine	Local inshore	105	29–40		16.6
Sansom & Sassoubre^50^	Freshwater mussel	Freshwater	Tap	147	22		17.8
Andruszkiewicz et al.^21^	Pacific chub mackerel	Marine	Local inshore	107	17		17.8
Sansom & Sassoubre^50^	Freshwater mussel	Freshwater	Tap	147	22		18.2
Andruszkiewicz et al.^21^	Pacific chub mackerel	Marine	Local inshore	107	17		18.2
Sigsgaard et al.^17^	Whale shark	Marine	Local inshore	105	29–43		18.7
Tsuji et al.^31^	Ayu sweetfish	Freshwater	River	131	10	7.5	19.6
Eichmiller et al.^14^	Common carp	Freshwater	Well	149	15		20.0
Tsuji et al.^31^	Common carp	Freshwater	River	78	10	7.5	20.5
Minamoto et al.^16^	Japanese sea nettle	Marine	Local inshore	151	17–20		21.1
This study	Common shore crab	Marine	Harbour	153	10	8	21.6
This study	Common shore crab	Marine	Harbour	153	15	8.5	23.5
Thomsen et al.^18^	Five-spined stickleback	Marine	Local inshore	101	15		23.7
Sansom & Sassoubre^50^	Freshwater mussel	Freshwater	Creek	147	22		23.9
This study	Shanny	Marine	Inshore	132	10	8	24.8
Eichmiller et al.^14^	Common carp	Freshwater	Dystrophic lake	149	15		25.2
This study	Shanny	Marine	Inshore	132	15	8.5	27.4
Sansom & Sassoubre^50^	Freshwater mussel	Freshwater	Tap	147	22		28.9
This study	Common shore crab	Marine	Offshore	153	10	8.1	31.6
Weltz et al.^19^	Maugean skate	Marine	Local inshore	331	4		34.7
This study	Common shore crab	Marine	Offshore	153	15	8.6	35.8
This study	Shanny	Marine	Offshore	132	10	8.1	38.9
Lance et al.^13^	Bighead carp	Freshwater	Deionised	190	30		42.7
This study	Shanny	Marine	Offshore	132	15	8.6	45.6
Eichmiller et al.^14^	Common carp	Freshwater	Eutrophic lake	149	5		47.5
Thomsen et al.^18^	European Flounder	Marine	Local inshore	104	15		51.7
Lance et al.^13^	Bighead carp	Freshwater	Deionised	190	20	8	61.6
Weltz et al.^19^	Maugean skate	Marine	Local inshore	331	4		63.0
Cowart et al.^15^	Antarctic icefish	Marine	Local inshore	70	−1		71.1
Sansom & Sassoubre^50^	Freshwater mussel	Freshwater	Tap	147	22		71.5
Lance et al.^13^	Bighead carp	Freshwater	Deionised	190	20	7	72.3
Lance et al.^13^	Bighead carp	Freshwater	Deionised	190	20	7.5	72.3
Lance et al.^13^	Bighead carp	Freshwater	Deionised	190	20		79.2
Strickler et al.^12^	Bullfrog	Freshwater	Tap	84	20	4	97.9
Lance et al.^13^	Bighead carp	Freshwater	Deionised	190	20	6.5	97.9
Strickler et al.^12^	Bullfrog	Freshwater	Tap	84	35	4	110.9
Strickler et al.^12^	Bullfrog	Freshwater	Tap	84	35	10	110.9
Strickler et al.^12^	Bullfrog	Freshwater	Tap	84	5	4	128.0
Strickler et al.^12^	Bullfrog	Freshwater	Tap	84	35	7	128.0
Strickler et al.^12^	Bullfrog	Freshwater	Tap	84	5	7	138.6
Strickler et al.^12^	Bullfrog	Freshwater	Tap	84	20	7	138.6
Strickler et al.^12^	Bullfrog	Freshwater	Tap	84	20	10	138.6
Lance et al.^13^	Bighead carp	Freshwater	Deionised	190	12		200.4
Lance et al.^13^	Bighead carp	Freshwater	Deionised	190	4		234.3
Strickler et al.^12^	Bullfrog	Freshwater	Tap	84	5	10	332.7

Summary of published eDNA degradation rates for marine and freshwater eukaryotes following Eichmiller et al. ^14^, but including invertebrates. Rows are sorted by half-life (hours) from low (fastest decay) to high (slowest decay). Half-lives are calculated from the published rate constant *K* (also referred to as *β*) with the equation $t_{\frac{1}{2}} = \frac{{ln\left( 2 \right)}}{k}$

Compared with freshwater, marine systems are generally characterised by higher salinity and ionic content, typically higher pH, and more stable temperatures. These are factors which have been shown to promote DNA preservation, and tend to correspond to the lowest observed degradation rates^5,14,23,25,30,31^. Our artificial spatial gradient varied from an offshore treatment with high pH and salinity to an inshore treatment with a lower salinity and a slightly lower pH. This was designed to capture the abiotic heterogeneity that could be expected across the Western English Channel region over the period of a year, a magnitude of variation that will apply to other coastal temperate locations. We found salinity to be a better predictor of eDNA decay than pH, and with salinity varying more between locations and pH varying more over seasons (Table 3), this agrees with the finding that the spatial signal was stronger than the temporal signal, and is reflected in the correlation matrix of the combined predictor-covariate model. The lack of a statistically significant difference over season may be due to the relatively low degree of variation in pH and temperature. Seawater pH measured in this experiment was between around 8 and 8.6, which may not have any direct impact on DNA hydrolysis, and likewise, temperature ranges in this temperate marine system (10–15 °C) were narrower than those typically studied in terrestrial systems (e.g. 5–35 °C^12^).

As well as abiotic factors engaging in DNA degradation via oxidisation and hydrolysis by depurination, biotic factors are also likely to play a major role in eDNA persistence dynamics via extracellular DNases produced by heterotrophic microbes^4,30^. While we found support for faster degradation rates in our inshore and mixed treatments (Fig. 3), this difference did not appear to be proportional to the quantity of inshore water used in the treatment—the two-third offshore treatment tended to be closer to the 100% inshore treatment than the 100% offshore treatment—suggesting that biotic rather than abiotic factors are of stronger influence. Salinity itself may not be therefore entirely responsible for the difference in decay rate, rather that it is associated with particular abundances or communities of microbes. Gilbert et al.^32^ reported that microbial community structure in the Western English Channel was highly dynamic seasonally. Free DNA is thought to represent an important organic phosphorus source in marine systems^29^, and seasonal phosphate limitation has been identified as a key driver of eDNA turnover rates over abiotic factors such as temperature, pH and salinity^26^. Therefore, the lack of seasonal difference in eDNA decay that we report may also be explained by organic phosphorous or carbon concentrations^14,26^.

Taken together with the survey of rates from the literature, this implies that abiotic and biotic factors are co-implicated in eDNA degradation. Assessing the covariance and contribution among these parameters is an area that needs to be addressed, along with more sophisticated analyses of microbial communities incorporating a greater degree of spatial replication.

A number of systematic biases were identified as being potentially problematic for our inferences. PCR inhibition in the samples from the inshore site could explain the faster degradation rates from that location. However, we assessed amplification efficiency of the qPCR in a serial dilution experiment, and these were near the expected 100% across treatments and season. Values well above 100% would indicate inhibition. Other studies have also indicated low instances of PCR inhibition when using kits with dedicated inhibitor removal steps such as the PowerWater kit that we used^33,34^.

Although not significantly different, we found that degradation rates were overall around 1.2 times faster in the crab assay than the shanny assay. This is most likely explained by differences in fragment length between the two assays (153 vs. 132 bp), with longer fragments being shown to decay at a faster rate than shorter fragments^22^. It was also noted that despite using a similar mass of crabs and shannys to create the eDNA, initial measured concentrations were roughly an order of magnitude lower in the crab assay (Fig. 1), perhaps indicating that the exoskeleton of the crustaceans, as well as their behaviour and breeding condition at particular times of the year may limit eDNA output^35^.

The treatment of qPCR non-amplifications in low-template analyses is an important source of error at the analytical stage. Due to the proportion of non-amplifications at the 192-h sample (0.56 in winter and 0.68 in summer)—i.e. outside of the experimental limit of quantification—and the influence of this time point in estimating the regression slopes, our eDNA decay model was sensitive to how these missing data were treated. Excluding them, or fixing them to the limit-of-detection value resulted in the effects of season and assay becoming statistically significant. However, treating the non-amplifications this way is problematic as these missing data are not randomly distributed; the missing values will tend to be from samples of lower concentrations, and therefore the remaining positive values will then become overestimated^36^. Our conservative approach was to follow Ellison et al.^37^ and fix their value, although we used the lowest detectable concentration of the assay (13.7 copies/L) rather than fixing the values at zero. Unfortunately, fixing values in this way is also problematic, creating a potential underestimate of concentration, and may interfere with the assumptions of linear regression. A better future strategy may be to avoid estimating decay rates from low copy-number time series, or to impute the missing data^36^.

Related to the issue of missing data is that of starting concentrations. Despite normalising each time sample as the proportion of the *t* = 0 starting concentration, we included in our model the initial value and found it to be a statistically significant predictor associated with faster degradation rates. The summer experiment and the crab assay had lower starting concentrations than the winter experiment and the shanny assay respectively (Fig. 1), but although the average crab-assay decay rate was faster than shanny, the average winter rates were faster than that of summer. Therefore, while they may not have influenced the results overall, a low starting concentration of eDNA resulted in the lower resolution of the summer crab experiment in particular, as qPCR quantification is increasingly stochastic and unreliable at low-template concentrations^38^.

In terms of implications for marine ecology, how do eDNA half-lives or decay rate constants relate to detectability of a given organism? As suggested by Sassoubre et al.^20^, reporting the duration of time until the detection limit is reached is misleading, as this value will depend upon the starting concentration of eDNA and the sensitivity of the assay; most studies use eDNA starting concentrations far higher than typical natural concentrations in order to generate reliable decay curves with less noise. Our negative biological controls provide an insight into natural concentrations. Sutton Harbour (our inshore treatment) is well populated with common shore crabs, and as expected, we recovered this species at approximate concentrations of 263 copies/L (winter) and 270 copies/L (summer). As the detection rate of the crab assay was 37% at 83 copies/L, and the eDNA half-life inshore was around 24 h, it is estimated that the chance of detection with three PCR replicates would be below the threshold after just two half-life periods (~48 h). However, we did detect eDNA in at least one qPCR replicate from this control at all time points up to 192 h (winter) and 48 h (summer), indicating that eDNA detectability will be difficult to predict at very low concentrations. Quantitative PCR is known to be more sensitive than standard PCR combined with metabarcoding^39^. Thomsen et al.^18^ estimated similar values of natural eDNA to ours (535 copies/L for flounder, 120 copies/L for stickleback), and a similar detection limit (63 copies/L). However, it must be noted that we did not consider the loss of DNA in the extraction process, which can be considerable with commercial kits that incorporate steps to remove PCR inhibitors^34,40^, or any loss of eDNA at the filtration stage, and therefore, real values are likely to be higher and comparisons among studies using different methodologies may be questionable.

Ultimately, how eDNA persists and moves through an environment can have important repercussions for making meaningful ecological inferences, and it is important to document and understand the patterns and processes involved^41,42^. The combined issues of degradation, transportation and dilution of eDNA are of particular importance in the marine environment, due to the effects of tides and large water volumes^9,18,43^. Fortunately, eDNA metabarcoding studies of marine systems have reported a strong local eDNA signal, either closely matching lists of expected fauna^18,44,45^ or reporting an expected turnover in diversity over short spatial or temporal scales^9,43,46^. Most evidence therefore points to eDNA surveys offering a contemporaneous representation of a community, even over the variation encountered on a daily tide^9^. However, there are cases where non-resident freshwater species have been detected in marine eDNA studies^47^, and while this source of error can easily be discarded as clearly a riverine input, currents transporting possibly co-occurring marine species eDNA may cause a less obvious source of systematic bias. These biases may become more serious when eDNA is used in applications beyond determining occurrence, for example to monitor the spread of marine invasive species^48^ or correlating with animal biomass estimates^49^. By incorporating eDNA degradation rates in different types of water body with oceanographic modelling of tidal currents, it will be possible to build well-informed predictive probability maps of organismal distribution^44,48,50^. Until these are available, to our knowledge, we show for the first time that it is reasonable to assume large variation in eDNA persistence according to local factors such as salinity gradients over relatively short local scales corresponding to marine environmental stability.

## Methods

### Assay design

Study species were the shanny (Teleostei: Blenniidae: *Lipophrys pholis*) and the common shore crab (Decapoda: Portunidae: *Carcinus maenas*). These species were chosen because they are abundant hardy organisms amenable to transport and experimental manipulation. Reference specimens of shanny (eight individuals) and shore crab (four individuals) were obtained from the Gann estuary, Pembrokeshire, Wales (51.715, −5.173). Using standard molecular methods, we obtained DNA barcodes (*COI*; 5′ mitochondrial cytochrome c oxidase I gene) for both species using the FishF1/R1 primer set^51^. Additional sequence data for crabs (149 individuals) were obtained from GenBank; no GenBank *COI* sequences were available for shanny. Primers and hydrolysis probes were designed using Primer3 v1.1.4^52,53^ under default settings adjusted to aim for an amplicon length between 50 and 170 bp. The resulting 12 candidate primer pairs were tested in silico for general specificity against a dataset of sequences from species present in the United Kingdom. To generate a list of fishes and Malacostraca recorded from the United Kingdom, we searched the Global Biodiversity Information Facility (https://www.gbif.org https://www.gbif.org) using the rgbif v0.9.9 package for R^54^. *COI* sequences for these species were then retrieved from GenBank and annotated using rentrez v1.2.1^55^ and traits v0.3.0.9310^56^. Each candidate primer pair was tested in an in silico PCR using MFEprimer v2.0^57^ using liberal settings (*k* = 5). The final primers were then chosen based on a combination of amplicon length, specificity and melting temperatures, and are reported in Supplementary Table 1. The reporter dye for the shanny assay was FAM, and for the crab assay HEX; both were quenched using BHQ.

### Experimental setup

The experiment was repeated twice, first in winter (water collected on 17 February, 2017) and once in late summer (water collected on 26 September, 2017). All treatments were set up in a dedicated temperature-controlled aquarium room held at temperatures consistent with natural seawater temperatures at that time of the year (10 °C, winter experiment; 15 °C, summer experiment). Animals were collected 2 days before the start of each experiment (also from the Gann estuary, Pembrokeshire) and placed in a separate and aerated holding tanks for each species (shanny, 50 L of synthetic seawater; crabs, 25 L). Approximately 300 g of animal mass per species were collected (winter, 24 shannys at 343 g of total weight, 18 crabs at 288 g; summer, 25 shannys at 316 g, 9 crabs at 304 g). All animals were euthanised after the experiment was completed, and were formalin fixed and 70% alcohol preserved as voucher specimens for a reference dataset. All experiments were carried out in accordance with the University of Bristol ethical approval (UIN reference UB/16/012).

A total of 24 aquariums at the University of Bristol Animal Services Unit were each filled with 9 L of experimental water. The tanks were initially mixed but not aerated and were maintained under 12 h of light/dark LED room lighting. Five experimental water treatments were carried out as follows: 100% offshore sea surface water—from herein referred to as 'offshore'—collected from Western Channel Observatory station E1 ~40 km from Plymouth, Devon, UK (50.033, −4.367; Supplementary Fig. 2); inshore urban water—from herein referred to as 'inshore'—collected from Sutton Harbour, Plymouth Sound, a site located between the estuaries of the rivers Plym and Tamar (50.370, −4.133; Supplementary Fig. 2); a two-thirds/one-third mixture of offshore to inshore water; a one-third/two-thirds mixture of offshore to inshore water; and synthetic seawater made using a proprietary aquarium salt mix. Each of the five treatments had four biological replicates (=20 tanks), plus four no-treatment controls (2× synthetic seawater, 1× offshore and 1× inshore.

After turning off aeration and allowing detritus to settle for an hour, 500 mL of eDNA-rich surface water from both the shanny and crab stock tanks was then added to each experimental tank at the start of the experiment. At each subsequent time point, eDNA was filtered from 600 mL of experimental tank water with a peristaltic pump and Sterivex 0.22-μm PES filters (Millipore part no. SVGP01050)^58^. Measurements were taken at six intervals from the same tanks (0, 12, 24, 48, 96 and 192 h), resulting in 144 filtered water samples (24×6). After being cleared of water, filters were frozen immediately at −20 °C. DNA was subsequently extracted from the Sterivex filters using the PowerWater DNA isolation kit (MoBio/Qiagen part no. 14900-100-NF) following manufacturers’ instructions, but with 50 μL of final elution volume. Extractions were carried out in a dedicated pre-PCR extraction room regularly decontaminated with 10% bleach and UV sterilisation.

Environmental covariates were also measured from each tank with a Hach HQ40D multimeter, and included salinity (conductivity in mS/cm), pH, and temperature at source. As a proxy for biological activity, we also recorded total background double-stranded DNA (dsDNA) concentration from 2 L of source water with a Qubit 3 fluorometer (ThermoFisher) assay (filtered and extracted in the same way as the experimental treatments).

### Quantitative PCR

Quantitative PCR reactions were conducted as per the manufacturer’s instructions, in multiplex, on a PCRmax Eco48 machine in 48-well plates of 5 μL per reaction, with ROX normalisation. Each reaction volume comprised 2.5 μL of mastermix (qPCRBIO Lo-Rox Probe mix; part no. PB20.21-05); 0.5 μL of shanny–crab primer-probe mix (optimised reaction concentration for shanny assay: 600 nM each primer, 200 nM probe; crab assay: 600 nM each primer, 300 nM probe); 1 μL of water and 1 μL of eDNA template. The cycling parameters comprised 3 min at 95 °C polymerase activation followed by 42 cycles of denaturation at 95 °C for 5 s and combined extension/annealing at 60 °C for 30 s.

Each plate of 48 reactions comprised: eight extracted water samples of the experimental tanks, with three technical replicates per sample (8 × 3 = 24 reactions); a six-fold standard-curve serial dilution of 1–1 million copies/μL, in triplicate (=21 reactions); and three no-template controls (=three reactions). To allow low-copy-number templates, an increased opportunity to amplify, PCRs were repeated a further three times for each sample when there was no amplification in any of the three initial technical replicates (excluding negative controls). The standard curve stock solutions were generated by PCR-amplifying and purifying tissue extractions of genomic DNA in a standard PCR using the primers in Supplementary Table 1, and were subsequently diluted and quantified using a Qubit assay, with the number of copies estimated at a standard dsDNA molar mass of 650 g^28^.

We tested for PCR inhibitors by performing triplicate qPCRs on three serial dilutions of the 0 h replicates from three treatments (synthetic, inshore, offshore) over both seasons (total 24 samples). If inhibitors were co-extracted, the cycle threshold (Ct) values at each tenfold dilution point would deviate from the expected increase of 3.3 PCR cycles, and therefore the expected efficiency values of 90–110%^28^.

### Analysis

Cycle threshold values and target DNA concentrations were calculated on the Eco48 machine software using the default settings, and converted from copies per reaction (=copies/μL given a 1-μL template volume) to copies/L of initial sample water (given a 600 mL filtration volume and a 50 μL elution volume). All amplifications were checked manually in the log plot view and any amplifications that crossed the baseline threshold, but that did not represent a clean, obviously exponentially increasing reaction, were excluded. The final eDNA concentrations for each sample were averaged over the technical replicates, with non-amplifications included as an arbitrarily low but non-zero value of 13.7 copies per litre of sample water (Ct = 38.5; the lowest concentration that the assay reliably detected).

Statistical analyses were conducted in R v3.5.1^59^. Decay of eDNA was modelled using a linear mixed-effects model as implemented in the lme function of nlme v3.1-137^60^. The response variable was natural log_*e*_ transformed eDNA concentration normalised as a proportion of starting concentration, i.e. the value at time *t* = *x* divided by the value at time *t* = 0. We specified time, treatment, season, assay, and the natural log of eDNA starting concentration as predictor variables (our fixed effects), while the individual tank used in each biological replicate was treated as a random effect. To minimise heteroscedasticity—i.e. the increasing variance of regression residuals over time—we excluded the normalised zero-hour (*t* = 0) data, which had no variance. The synthetic water control was also excluded from the main model—this was a control for reference rather than to investigate its biological effect—and decay rates for this subset were calculated separately (following the same procedure as outlined below).

We determined the optimal model to fit our data according to the procedure of Zuur et al.^61^. We started with a full model containing all fixed effects and their interactions, and determined the optimal variance weighting for different treatment–season–assay combinations by AIC comparison (given by the form weights = varIdent(form = 1|treatment*season)). We then determined the optimal random structure for the full model with this variance weighting by AIC comparison (given by the form random = 1 + time|tank). Finally, we determined the optimal fixed effects structure using the 'drop1' approach and specifying method = ’ML’ until all terms in the model were statistically significant. We switched to method = ’REML’ and performed model validation to ensure that the model residuals were approximately normal and homogeneously distributed (see Supplementary Fig. 3). The fixed effects structure and output for the full model and the optimal model are also presented in Supplementary Note 1.

The first-order decay-rate constant *k* for each treatment–season–assay combination was calculated from the estimated marginal mean of regression slopes using the emtrends function of emmeans v1.2.3^62^. To test the importance of predictor variables on the degradation rate, pairwise post hoc Tukey tests were carried out on the marginal mean regression slopes, again using emmeans. To explore the environmental covariates we constructed a simple lme model with time, assay, pH, conductivity, natural log transformed starting concentration, and background DNA concentration as fixed effects, and tank as a random effect. For this model, we excluded the treatment and season predictors—which were deliberately chosen for their heterogeneity—as we assumed these to be correlated with the environmental covariates. We additionally included them in a combined model to estimate the degree of correlation between the predictors and covariates.

### Code availability

The code generated during and/or analysed during the current study is available in the Figshare repository^65^, 10.6084/m9.figshare.7111376.v1.

## Electronic supplementary material

Supplemental Information

## Data Availability

The datasets generated during and/or analysed during the current study are available in the Figshare repository^65^, 10.6084/m9.figshare.7111376.v1. New sequence data generated here were deposited in the GenBank nucleotide archive under the accessions MH931374:MH931388.
