# Supplementary material for: Persistence of environmental DNA in marine systems
Source: Commun Biol. 2018 Nov 5;1:185. doi: 10.1038/s42003-018-0192-6 (PMC6218555; doi:10.1038/s42003-018-0192-6)
Supplement: Supplementary file 1 — Supplemental Information [file 42003_2018_192_MOESM1_ESM.pdf]

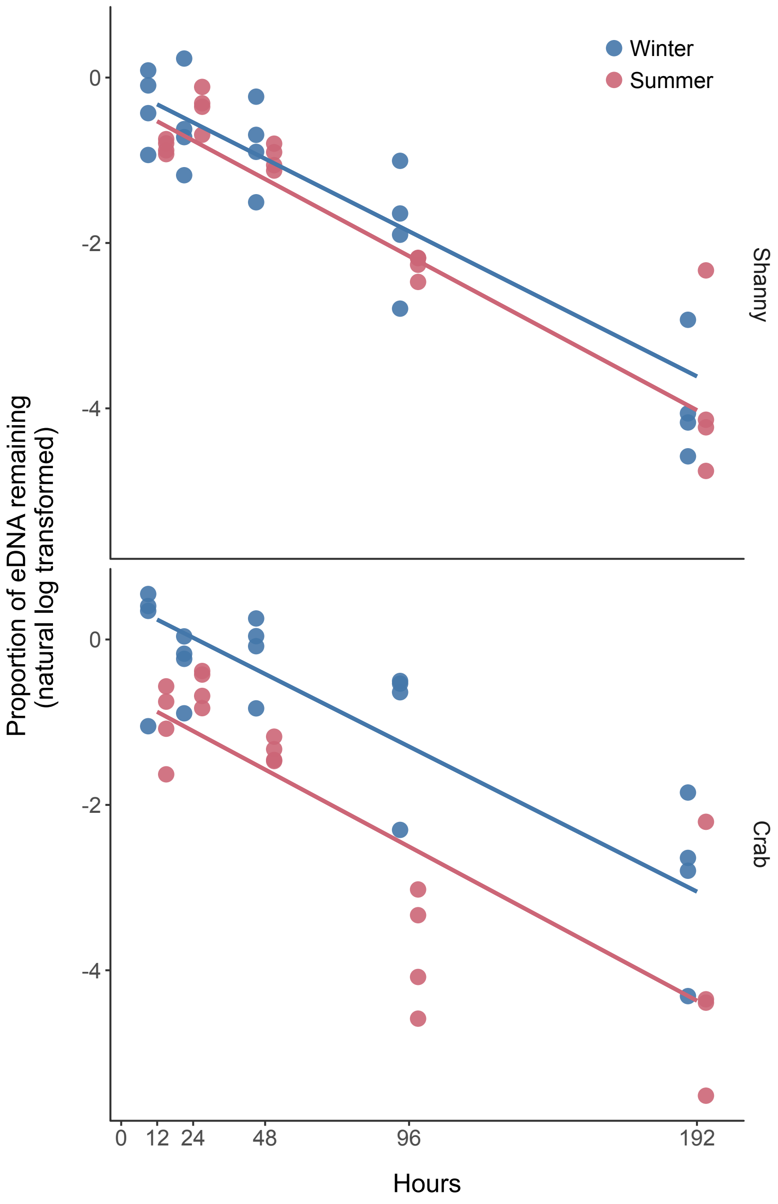

Supplementary Figure 1: Synthetic water control. Environmental DNA decay in the synthetic water treatment control over 192 hours, two seasons (summer and winter), and two species (shanny and common shore crab assays). The response variable is natural $\log_e$ transformed eDNA concentration normalised as a proportion of starting concentration, i.e. the value at time $t = x$ divided by the value at time $t = 0$. Zero hour data at $t = 0$ were subsequently excluded after proportions were calculated. Trend lines show fitted linear regression values from the optimal linear mixed-effects model (specific for the synthetic control).

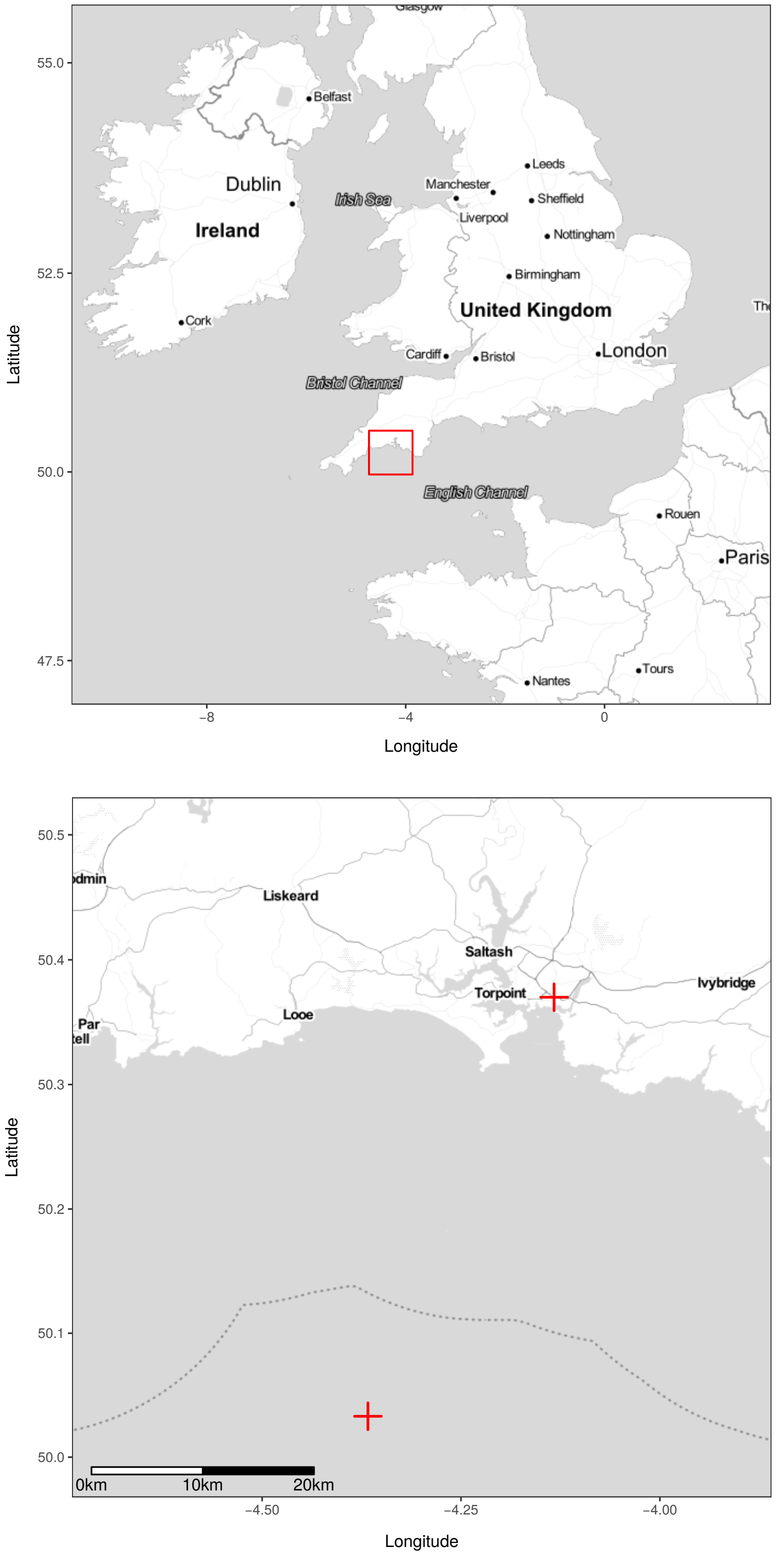

Supplementary Figure 2: Study site map. Map of study sites in relation to the Western English Channel. Upper panel shows position of study site in reference to the UK, Republic of Ireland, and France. Lower panel shows water collection sites as marked with red crosses. Inshore site to the north (50.370, −4.133), offshore site to the south (50.033, −4.367). Dotted line shows limit of territorial waters of the UK.

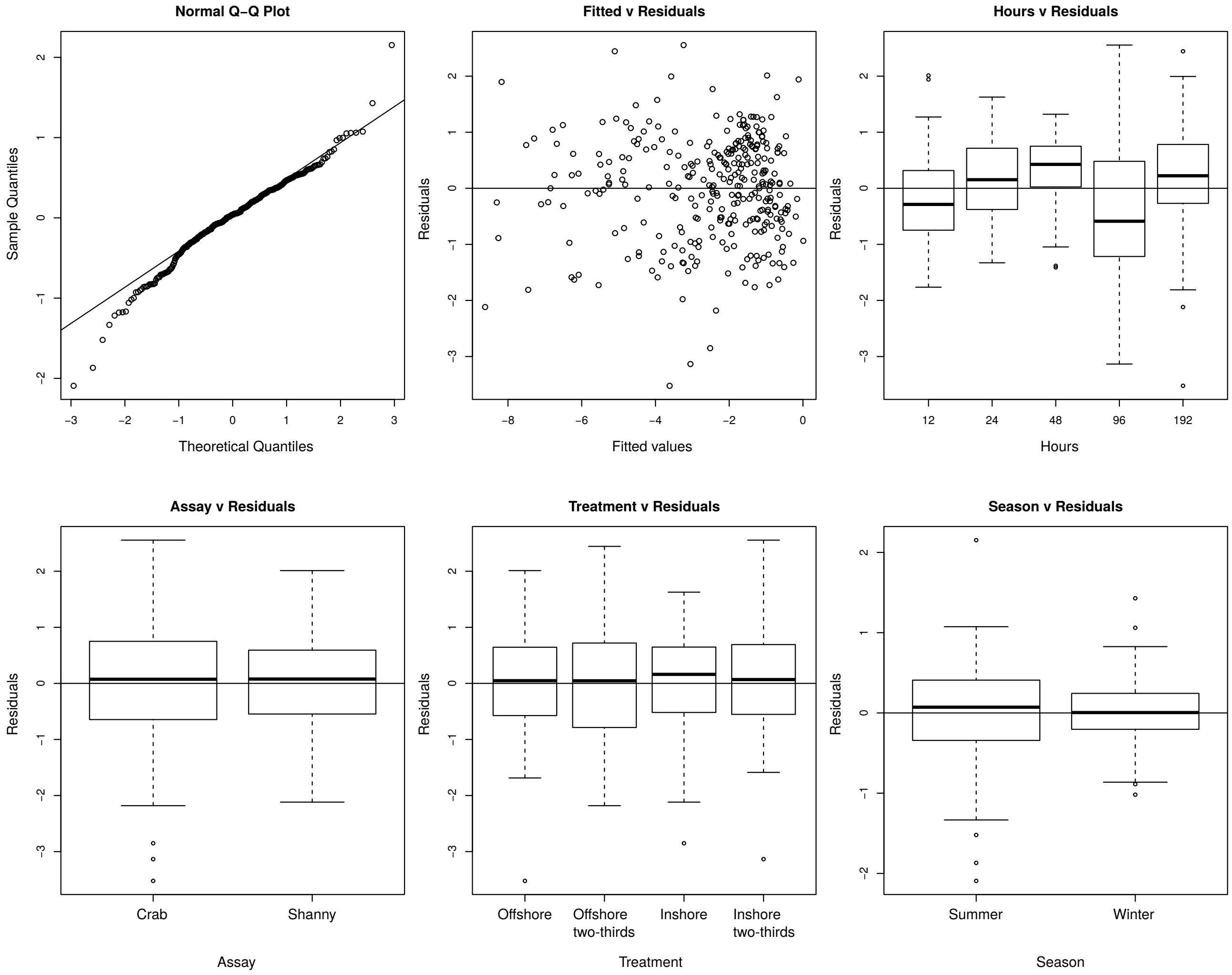

Supplementary Figure 3: Model diagnostics. Residuals plots for the optimal (post model selection) linear mixed-effects (lme) model. See below for model summary, plus the fixed effects structure of the full model before model selection.

Target species	Amplicon length	Location	Reporter dye	Primer	Oligonucleotide sequence (5′–3′)	Oligo. T_m (°C)
Lipophrys pholis	132 bp	5,800	FAM	Forward	TTTTAGGGGCAATCAACTTCA	59.6
				Reverse	GGACTGGGAGGGATAAAAGAA	59.4
				Probe	CGCCCCTCTTTGTTTGGGCTG	70.0
Carcinus maenas	153 bp	5,783	HEX	Forward	AGCCGGGGTTTCTTCTATTTT	60.3
				Reverse	GCTAAAACCGGCAACGATAAT	60.3
				Probe	TCAATATGCGTTCTTTCGGCATGACA	70.1

Supplementary Table 1: Primers and probes. Primers for two eDNA assays (partial *COI* gene): *Lipophrys pholis* (shanny) and *Carcinus maenas* (common shore crab). "Location" refers to the starting position of the forward primer in relation to the mouse mitochondrial genome AY172335.1.

Supplementary Note 1: Model output. R model output from the linear mixed-effects (lme) model, showing fixed effects structure before model selection, and estimated parameters of the final optimal model.

```
------------>>> FULL EDNA DEGRADATION FIXED EFFECTS MODEL BEFORE MODEL SELECTION <<<------------

log(prop0) ~ hours + assay + season + treatment + log(startConc) +
    hours:assay + hours:season + hours:treatment + hours:log(startConc) +
    assay:season + assay:treatment + assay:log(startConc) + season:treatment +
    season:log(startConc) + treatment:log(startConc) + hours:assay:season +
    hours:assay:treatment + hours:assay:log(startConc) + hours:season:treatment +
    hours:season:log(startConc) + hours:treatment:log(startConc) +
    assay:season:treatment + assay:season:log(startConc) + assay:treatment:log(startConc) +
    season:treatment:log(startConc) + hours:assay:season:treatment +
    hours:assay:season:log(startConc) + hours:assay:treatment:log(startConc) +
    hours:season:treatment:log(startConc) + assay:season:treatment:log(startConc) +
    hours:assay:season:treatment:log(startConc)


------------>>> FINAL EDNA DEGRADATION MODEL OUTPUT AFTER MODEL SELECTION <<<------------

Linear mixed-effects model fit by REML
 Data: tab.tmp
       AIC      BIC    logLik
  663.1723 781.1555 -299.5861

Random effects:
 Formula: ~1 + hours | tank
 Structure: General positive-definite, Log-Cholesky parametrization
            StdDev      Corr
(Intercept) 0.189454188 (Intr)
hours       0.004461309 -0.867
Residual    0.667719448

Variance function:
 Structure: Different standard deviations per stratum
 Formula: ~1 | treatment * season
 Parameter estimates:
harbour-twothirds*summer           e1-full*summer      e1-twothirds*summer      harbour-full*summer      e1-twothirds*winter           e1-full*winter harbour-twothirds*winter      harbour-full*winter
               1.0000000                0.7945787                1.3193067                0.7004891                0.6994865                0.4092634                0.8377049                0.4136670

Fixed effects: log(prop0) ~ hours + assay + season + treatment + log(startConc) + hours:assay + hours:season + hours:treatment + hours:log(startConc) + assay:season + assay:treatment + season:treatment

                                              Value Std.Error  DF   t-value p-value
(Intercept)                               3.1487863 1.0248750 271  3.072361  0.0023
hours                                     0.0124778 0.0106830 271  1.168011  0.2438
assayshanny-fam                           1.5440209 0.3377257 271  4.571819  0.0000
seasonwinter                              1.1420181 0.2239594  24  5.099219  0.0000
treatmente1-twothirds                     0.1555133 0.2455581  24  0.633306  0.5325
treatmentharbour-full                     0.4973698 0.1880085  24  2.645464  0.0142
treatmentharbour-twothirds                0.2200372 0.2223112  24  0.989771  0.3322
log(startConc)                           -0.4665564 0.1190195 271 -3.919999  0.0001
hours:assayshanny-fam                     0.0041436 0.0035651 271  1.162244  0.2462
hours:seasonwinter                       -0.0026011 0.0025454 271 -1.021902  0.3077
hours:treatmente1-twothirds              -0.0107489 0.0025998 271 -4.134454  0.0000
hours:treatmentharbour-full              -0.0101294 0.0024285 271 -4.171057  0.0000
hours:treatmentharbour-twothirds         -0.0107952 0.0025630 271 -4.211996  0.0000
hours:log(startConc)                     -0.0027387 0.0012426 271 -2.204011  0.0284
assayshanny-fam:seasonwinter             -0.4603269 0.1233594 271 -3.731591  0.0002
assayshanny-fam:treatmente1-twothirds    -0.7976788 0.1517173 271 -5.257665  0.0000
assayshanny-fam:treatmentharbour-full    -0.9907321 0.1099532 271 -9.010486  0.0000
assayshanny-fam:treatmentharbour-twothirds -0.7599429 0.1584459 271 -4.796231  0.0000
seasonwinter:treatmente1-twothirds        0.1339314 0.2268558  24  0.590381  0.5605
seasonwinter:treatmentharbour-full       -0.5585427 0.1741384  24 -3.207465  0.0038
seasonwinter:treatmentharbour-twothirds  -0.3183551 0.2115504  24 -1.504866  0.1454

Correlation:
                                         (Intr) hours  assys- ssnwnt trtm1- trtmnthrbr-f trtmnthrbr-t lg(sC) hrs:s- hrs:ss hrs:1- hrs:trtmnthrbr-f hrs:trtmnthrbr-t hr:(C) assy-: as-:1-
hours                                    -0.666
assayshanny-fam                           0.888 -0.670
seasonwinter                              0.616 -0.491  0.718
treatmente1-twothirds                     0.025  0.023  0.121  0.260
treatmentharbour-full                    -0.162  0.118 -0.010  0.246  0.401
treatmentharbour-twothirds               -0.037  0.039  0.093  0.278  0.351  0.448
log(startConc)                           -0.989  0.665 -0.923 -0.703 -0.097  0.064       -0.048
hours:assayshanny-fam                    -0.640  0.955 -0.703 -0.529 -0.023  0.049       -0.017        0.661
hours:seasonwinter                       -0.430  0.619 -0.480 -0.631  0.024  0.023       -0.006        0.474  0.684
hours:treatmente1-twothirds               0.036 -0.083 -0.014 -0.030 -0.541 -0.280       -0.238        0.014  0.020 -0.012
hours:treatmentharbour-full               0.080 -0.154  0.026 -0.018 -0.229 -0.587       -0.254       -0.025 -0.038 -0.018  0.470
hours:treatmentharbour-twothirds          0.046 -0.099 -0.008 -0.044 -0.218 -0.285       -0.558        0.008  0.011  0.019  0.446  0.478
hours:log(startConc)                      0.660 -0.982  0.687  0.542  0.024 -0.049        0.018       -0.677 -0.980 -0.699 -0.021  0.038           -0.011
assayshanny-fam:seasonwinter              0.332  0.070  0.016 -0.041  0.048 -0.031       -0.020       -0.294  0.069  0.049  0.001 -0.003            0.001           -0.071
assayshanny-fam:treatmente1-twothirds    -0.020 -0.008 -0.149 -0.025 -0.314 -0.101       -0.091        0.039 -0.008 -0.006  0.000  0.000            0.000            0.008 -0.023
```

```
assayshanny-fam:treatmentharbour-full      -0.078 -0.004 -0.261 -0.080 -0.126 -0.280       -0.131        0.106 -0.009 -0.003  0.000  0.000            0.000            0.005 -0.018  0.363
assayshanny-fam:treatmentharbour-twothirds  0.028  0.000 -0.140 -0.029 -0.076 -0.102       -0.356       -0.004  0.000  0.000  0.000  0.000            0.000            0.000  0.118  0.246
seasonwinter:treatmente1-twothirds         -0.073  0.028 -0.107 -0.369 -0.719 -0.270       -0.243        0.122  0.029 -0.021  0.100  0.032            0.032           -0.030 -0.062  0.008
seasonwinter:treatmentharbour-full          0.168 -0.045  0.090 -0.340 -0.265 -0.664       -0.301       -0.102 -0.034 -0.019  0.040  0.074            0.042            0.035  0.047 -0.006
seasonwinter:treatmentharbour-twothirds    -0.022  0.016 -0.065 -0.365 -0.241 -0.293       -0.623        0.075  0.020 -0.019  0.036  0.035            0.056           -0.021 -0.037  0.005
                                           assyshnny-fm:trtmnthrbr-f assyshnny-fm:trtmnthrbr-t ssn:1- ssnwntr:trtmnthrbr-f
hours
assayshanny-fam
seasonwinter
treatmente1-twothirds
treatmentharbour-full
treatmentharbour-twothirds
log(startConc)
hours:assayshanny-fam
hours:seasonwinter
hours:treatmente1-twothirds
hours:treatmentharbour-full
hours:treatmentharbour-twothirds
hours:log(startConc)
assayshanny-fam:seasonwinter
assayshanny-fam:treatmente1-twothirds
assayshanny-fam:treatmentharbour-full
assayshanny-fam:treatmentharbour-twothirds  0.346
seasonwinter:treatmente1-twothirds          0.020                    -0.001
seasonwinter:treatmentharbour-full         -0.016                     0.001                     0.383
seasonwinter:treatmentharbour-twothirds     0.012                     0.000                     0.344  0.418

Standardized Within-Group Residuals:
        Min          Q1         Med          Q3         Max
-3.52235281 -0.57431069  0.07550778  0.69096561  2.55448056

Number of Observations: 316
Number of Groups: 32


                     numDF denDF   F-value p-value
(Intercept)              1   271 2761.7905  <.0001
hours                    1   271  701.0155  <.0001
assay                    1   271  403.6088  <.0001
season                   1    24   14.3714  0.0009
treatment                3    24   39.5428  <.0001
log(startConc)           1   271   96.4602  <.0001
hours:assay              1   271   25.9574  <.0001
hours:season             1   271   12.2511  0.0005
hours:treatment          3   271    8.6765  <.0001
hours:log(startConc)     1   271    4.9504  0.0269
assay:season             1   271   11.9616  0.0006
assay:treatment          3   271   29.9246  <.0001
season:treatment         3    24    4.8523  0.0089
```
